# Development of a quantitative method to evaluate pedicle screw loosening after spinal instrumentation using digital tomosynthesis

**DOI:** 10.1186/s12891-022-05316-7

**Published:** 2022-04-15

**Authors:** Kentaro Mataki, Yuki Hara, Eriko Okano, Katsuya Nagashima, Hiroshi Noguchi, Yosuke Shibao, Kousei Miura, Hiroshi Takahashi, Toru Funayama, Masao Koda, Masashi Yamazaki

**Affiliations:** Department of Orthopedic Surgery, Faculty of Medicine, University of Tsukuba, 1-1-1 Tennodai, Tsukuba, Ibaraki 305-8575 Japan

**Keywords:** Pedicle screw loosening, Digital tomosynthesis, Displacement angle, Quantitative evaluation

## Abstract

**Background:**

In general, the diagnosis of pedicle screw (PS) loosening is evaluated qualitatively based on the presence of a radiolucent area around the implant wider than 1 mm on plain radiographs and computed tomography (CT). Digital tomosynthesis is a novel imaging technology that can acquire reconstructed tomographic images of patients in different postures with relatively low radiation. In this study, PS loosening is evaluated quantitatively by measuring the PS displacement angle in the vertebrae using digital tomosynthesis.

**Methods:**

We evaluated 41 patients who underwent posterior spinal fusion surgery using PS. The 72 pedicle screws at the cranial end of the fused segments were evaluated. The patients were divided in two groups, one with PS loosening (7 patients, 12 screws) and the other without PS loosening (34 patients, 60 screws), based on conventional CT findings. All patients underwent tomosynthesis in two different postures during a single CT session.

**Results:**

The displacement angles of the PS in patients in a lying position and in a standing position were measured using selected slices of the same cross-sectional view from digital tomosynthesis. The displacement angle was significantly greater in the PS loosening group (5.7°) than in the group without PS loosening (0.6°) (*p*<0.01). Based on the ROC analysis, the optimal cut-off value of the PS displacement angle for identification of loosened screws was 1.7° with a sensitivity of 100% and specificity of 93% (AUC = 0.98).

**Conclusions:**

This new method using digital tomosynthesis has the potential to aid diagnosis of PS loosening quantitatively and more accurately than conventional evaluations.

## Background

As the number of elderly people grows, spinal fusion surgeries for vertebra fracture, degenerative lumbar spondylolisthesis, and adult spine deformities are becoming more common. The pedicle screw (PS) is used as a rigid fixator for spinal fusion.

Several reports have described PS loosening as a complication of spinal fusion surgery. PS loosening causes reduced fixation and significant clinical problems. PS loosening has been reported to be related to various factors such as bone density and screw morphology. The rate of PS loosening has been reported to range from 0.8 to 27% and may exceed 50% in patients with osteoporosis [1–3]. For these reasons, fusion devices used for osteoporotic patients, which are of great interest to the research community [4, 5], require specific attention and design enhancement to improve the strength of the bone-screw interface.

For quality evaluation of a developed product, it is necessary to establish a quantitative method for evaluating PS loosening. Furthermore, early detection of PS loosening after surgery might save the patient not only painful spasms but also possible serious neural damage or screw breakage and revision surgery. A key problem for the investigation of screw loosening is the assessment of whether a screw is loosened or not, which is usually based on a radiological approach [6]. The diagnosis of PS loosening has been based on qualitative assessments from plain radiographs or computed tomography (CT) and is based on the presence of the halo sign, which is a radiolucent line around the implant wider than 1 mm^2^. There are few quantitative methods to evaluate PS loosening [3, 5].

PS loosening is caused by displacement of the PS in the vertebra. Therefore, PS loosening can be quantitatively measured by measuring PS displacement. Emin et al. reported a quantitative method to diagnose PS loosening by measuring change in angle between the PS axis and the end plate of the vertebra on plain radiographs at different postoperative timepoints [3].

Digital tomosynthesis is a new radiographic technique that can produce an arbitrary number of section images of a patient from a single pass. Digital tomosynthesis is used to acquire tomographic images with lower radiation than CT and results in better visibility of structures such as the vertebral trabeculae. Furthermore, examinations by digital tomosynthesis with the patients lying and standing can be made easily, facilitating dynamic examination. One of the other major advantages of digital tomosynthesis is that it can provide the same slice images while the patient is lying and standing because of reconstruction within − 20° to 20° against the operating table. This has been extremely difficult when using plain radiographs.

In this study, we devised a new quantitative method to diagnose PS loosening using digital tomosynthesis with the patient in different positions.

## Materials and methods

### Clinical study design

This study was performed at the University of Tsukuba Hospital, and was approved by the responsible ethics committee at the University of Tsukuba Hospital.

The study design was retrospective with cross-sectional analysis of PS loosening on CT (Brilliance iCT Elite, Koninklijke Philips N.V.Co, Netherlands) and digital tomosynthesis. Before enrollment, written informed consent was obtained from all the patient participants.

### Patients and the evaluated screws

The study included 41 consecutive patients (male 21, female 20, average age 67.5 years old) who underwent thoracolumbar spinal fusion surgery with PS in our institution between November 2017 and May 2020. All patients underwent CT and digital tomosynthesis within 1 month interval at the various time point after surgery (1 week to 20 months after surgery).

The incidence of PS loosening has been reported to occur near the ends of the fusion levels [7]. Therefore, in this study, 72 pedicle screws at the cranial end of the fused segments were evaluated using CT and digital tomosynthesis. Screws that could not be visualized in the same slice as the screw axis in supine and upright positions in the digital tomosynthesis were excluded. The number of 10 pedicle screws (13%) were excluded in the procedure due to issues with the plane of screw axis.

### Evaluation of PS loosening by CT

**Fig. 1:**
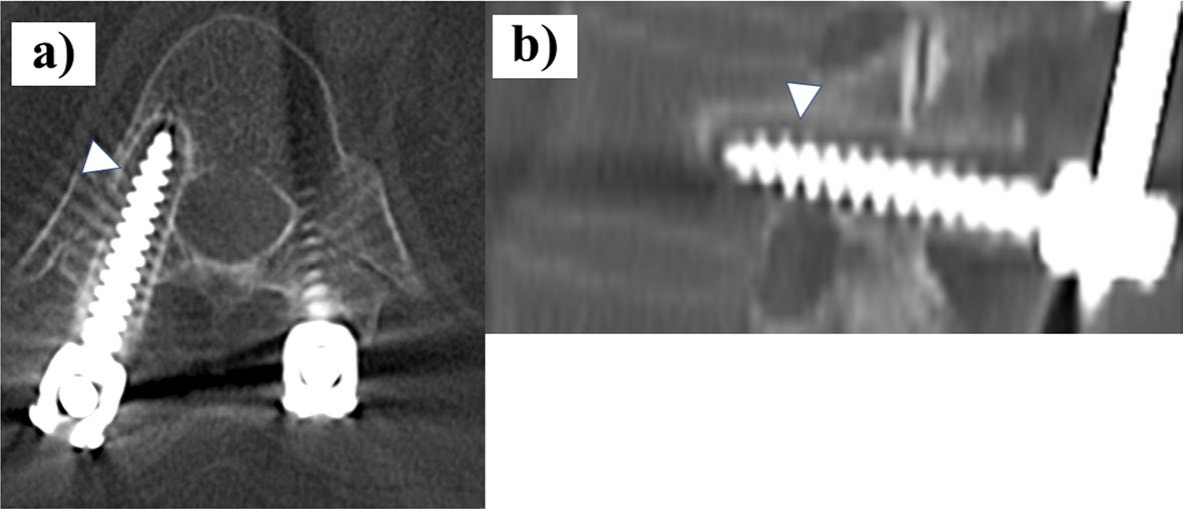
Axial (**a**) and sagittal (**b**) CT scan of a patient subjected to thoracolumbar posterior fixation showing a radiolucent area and the halo sign, indicative of screw loosening, indicated by the white arrowheads

All CT scans were performed at the University of Tsukuba Hospital. Screw loosening was determined on postoperative CT scans using conventional standard radiologic criteria, with or without a radiolucent area greater than 1 mm wide or the halo sign [2, 8, 9]. We divided the patients into two groups: one of seven patients (12 pedicle screws) who were diagnosed with pedicle screw loosening because of a radiolucent area wider than 1 mm or the presence of the halo sign on CT (Fig. 1), and the other of 34 patients (60 pedicle screws) without PS loosening on CT.

### Evaluation of PS displacement angle within the vertebrae by digital tomosynthesis in different postures

**Fig. 2:**
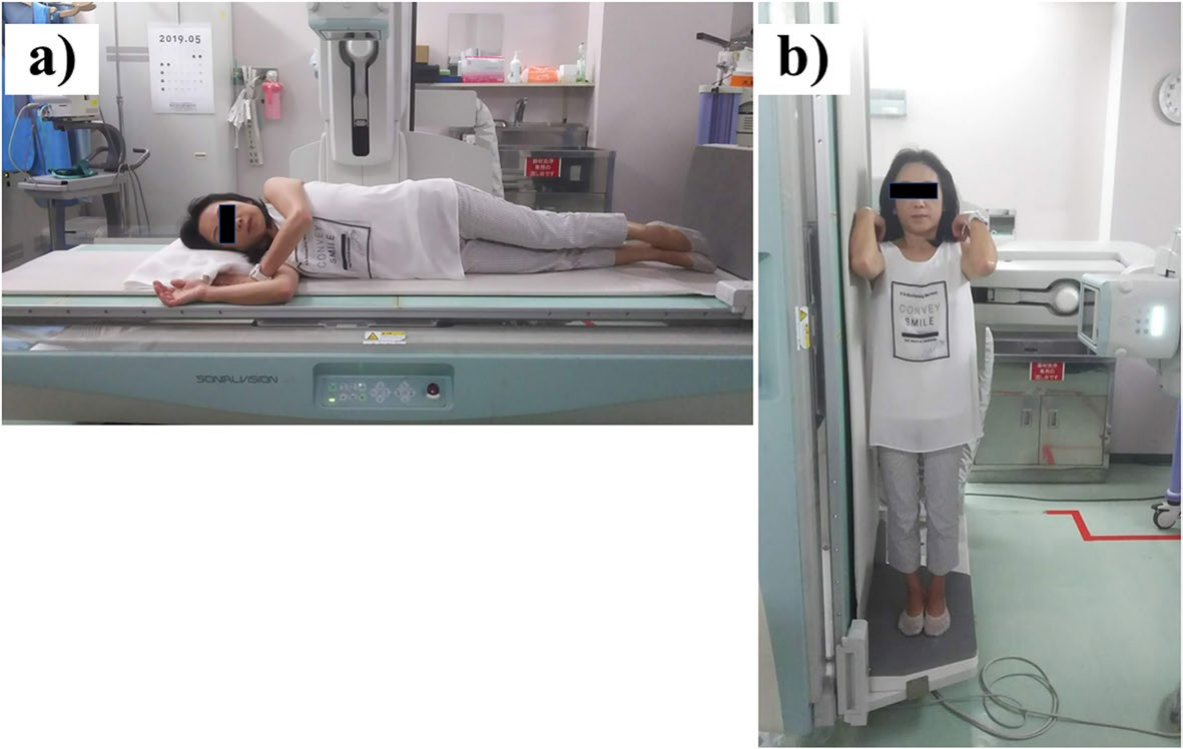
The examination using digital tomosynthesis (Shimazu SONIALVISION G4 Series) in different postures. **a** lateral lying position (**b**) standing position

The examination of the digital tomosynthesis (SONIALVISION G4, Shimazu Co, Japan) image in different postures was performed at the University of Tsukuba Hospital. In the first step, patients were placed in a lateral lying position on the operating table and a lateral view of the thoracolumbar spine was obtained (Fig. 2a). Then, patients were placed in a standing position by tilting the operating table and a lateral view of the thoracolumbar spine was obtained in the standing position (Fig. 2b).

Those patients who were unable to maintain the standing position safely were placed in a sitting position. Two out of 41 patients underwent tomosynthesis in the sitting position instead of the standing position.

**Fig. 3:**
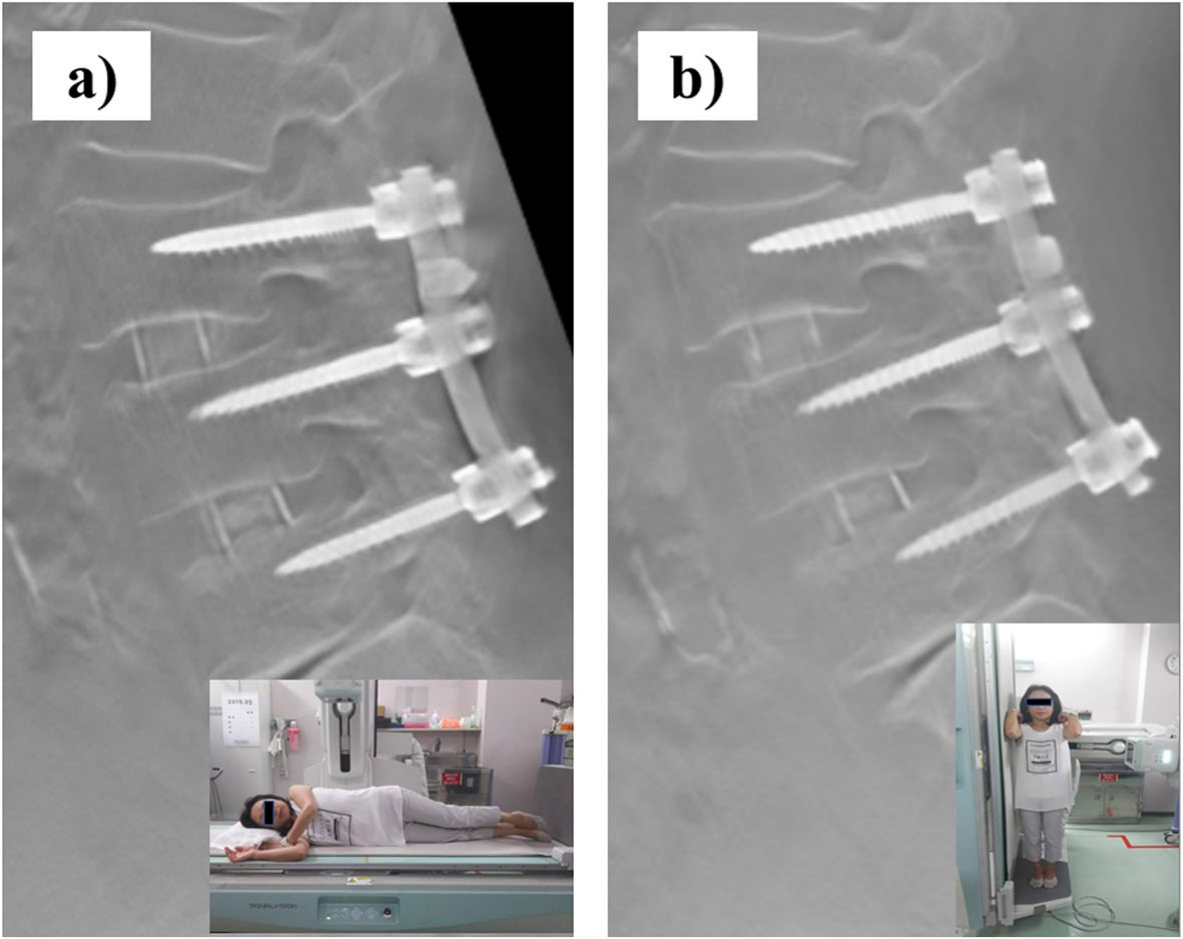
The same lateral plane between (**a**) lying and (**b**) standing positions from digital tomosynthesis without PS loosening. The plane of evaluation depicted both the full length of the PS axes at the cranial end of the fused segments and the connecting rod

After the examinations, the images were reconstructed at a workstation (SIDE STATION i3). The accuracy of coincidence of the same lateral plane between lying and standing positions was defined by the plane that depicted both the full length of the PS axes and the connecting rod. (Fig. 3).

**Fig. 4:**
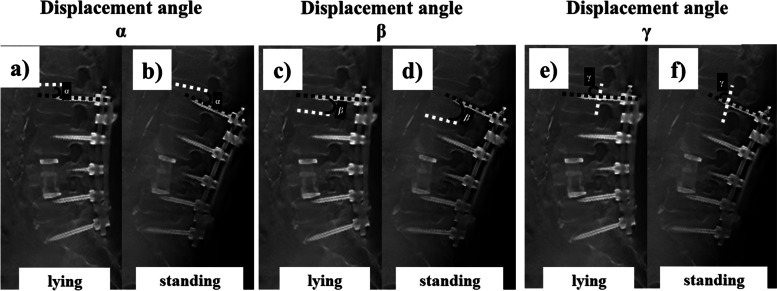
The same lateral plane between lying and standing positions from digital tomosynthesis in the PS loosening group. The displacement angle α (black solid line) between the PS axis (black dotted line) and the cranial endplate (white dotted line) of the same vertebra in the lying (**a**) and standing (**b**) position. The displacement angle β (black solid line) between the PS axis (black dotted line) and the caudal endplate (white dotted line) of the same vertebra in the lying (**c**) and standing (**d**) position. The displacement angle γ (black solid line) between the PS axis (black dotted line) and the posterior wall (white dotted line) of the same vertebra in the lying (**e**) and standing (**f**) position

Three different angle measurements (α, β, γ) were performed and compared for their capability to discriminate PS loosening between the lying position and the standing position: (1) the angle α between the PS axes and the cranial endplate of the same vertebra; (2) the angle β between the PS axes and the caudal endplate of the same vertebra; (3) the angle γ between the PS axes and the posterior wall of the same vertebra (Fig. 4). The fixed point was determined by enlarging the image up to 3200 times using ImageJ (the U.S. National Institutes of Health, USA) and applying the pixel selection method [10].

The PS displacement angle was defined as the average of the change in the three angles between the lying and the standing positions.

In the second step, the PS displacement angles were compared between the groups with PS loosening and without PS loosening that were defined by CT diagnosis.

In the third step, a receiver operating characteristics (ROC) curve analysis was performed and the area under the curve (AUC) was assessed to identify optimal cut-off values for the displacement angle that discriminated between patients with and without screw loosening. The sensitivity and specificity of the optimal cut-off values were calculated.

### Statistical analysis

Nominal patient characteristics between the groups were compared using the Chi-square test. The Mann–Whitney U test was used when studying the association of categorical and continuous variables. All statistical analyses were conducted using JMP software package, version 10.0.2 (SAS Institute, Inc., Cary, NC, USA). Significance was set at *p* < 0.05.

## Results

The seven patients (12 pedicle screws) were diagnosed with pedicle screw loosening because of a radiolucent area wider than 1 mm or the presence of the halo sign on CT. The rate of screw loosening at the cranial end of the fused segments was 16% in this study.

No significant differences in the average age or number of fused segments between patient groups were observed (Table 1). In the PS loosening group diagnosed by CT, the average PS displacement angle was 5.7° ± 5.1. In the group without PS loosening, the average PS displacement angle was 0.6° ± 0.5. The PS displacement angle was significantly different between the two groups (*p* < 0.01). There was no significant difference in the examination period between patient groups (Table 1).

**Table 1:** Demographic characteristics of the groups

	PS loosening (+)	PS loosening (−)	***P***
N	7	34	
% female	14	55	
age	69.8 ± 8.7	67.0 ± 10.9	0.23
Average N of fused segments of	5.5 ± 3.1	4.9 ± 4.1	0.51
Average of the examination period after surgery (weeks)	21.7	15.1	0.06
Displacement angle	5.7 ± 5.1	0.6 ± 0.5	**< 0.00001**
N of revision surgeries	2	0	

**Fig. 5:**
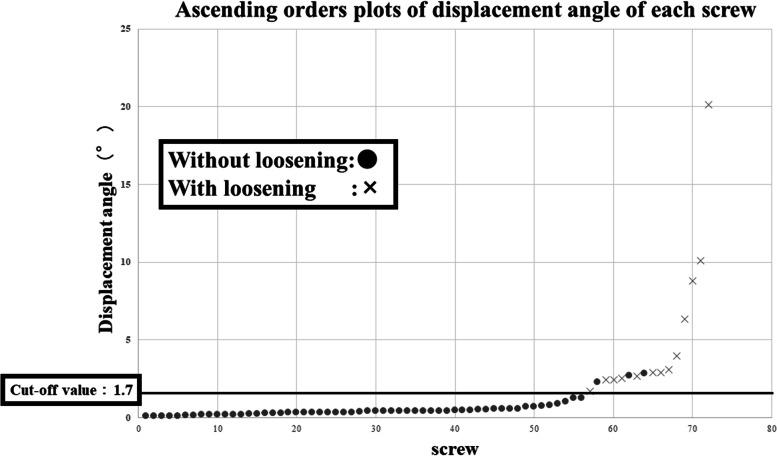
Ascending orders plot of displacement angle of each screw drawn as a continuous variable curve. The plots with screw loosening are indicated by a cross (×) and those without loosening are indicated by a circle (●). Screw loosening was associated with large displacement angles. There is no loosening screw with less displace angle than cut-off value (solid line)

An ascending orders plot of the displacement angle of each screw was drawn as a continuous variable curve. Screw loosening was associated with large displacement angles (Fig. 5).

**Fig. 6:**
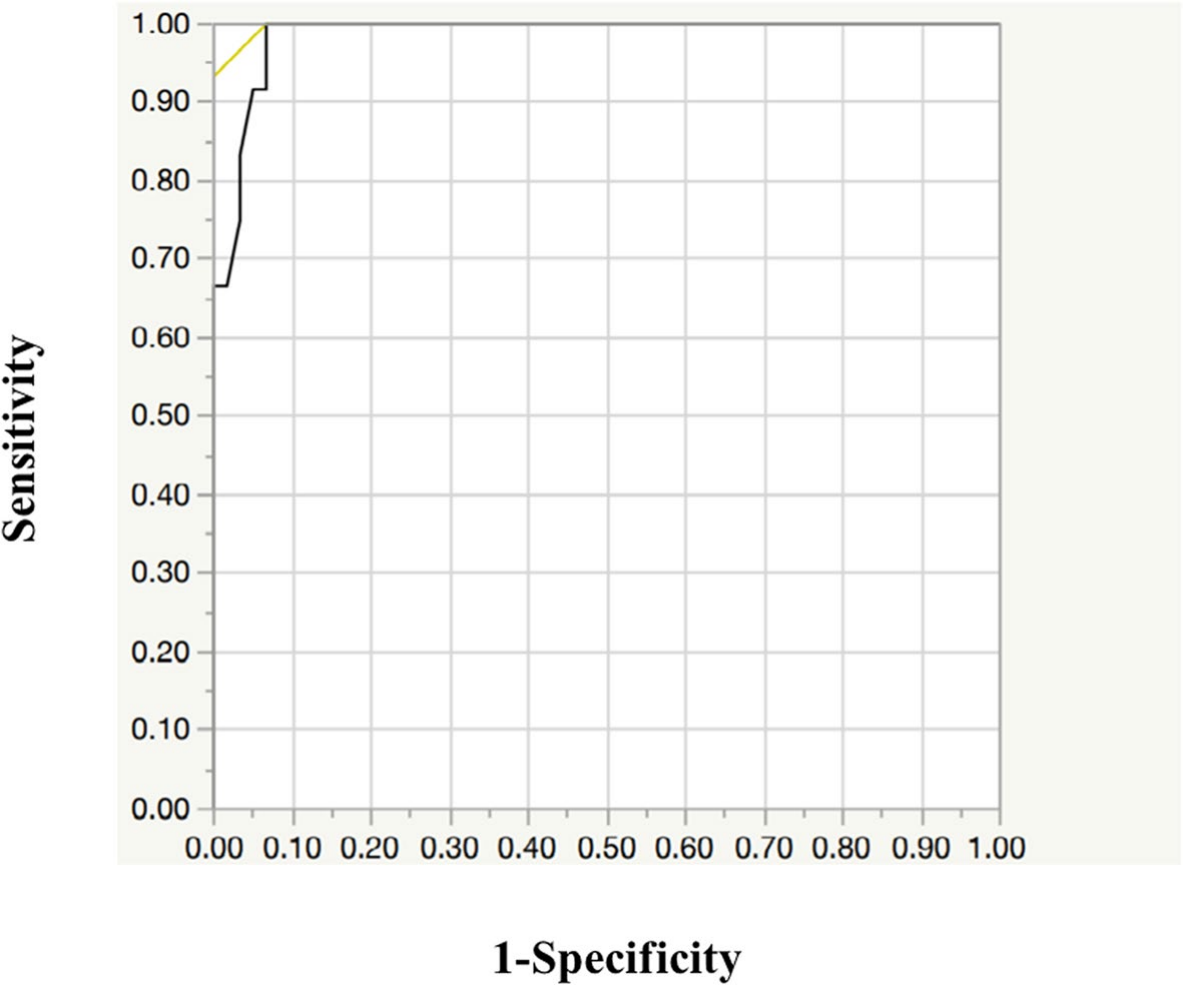
ROC curve analysis for the identification of an optimal cut-off value discriminating between those with and without PS loosening on CT images

Based on the ROC analysis, the optimal cut-off value of the PS displacement angle for identification of loosened screws was 1.7° with a sensitivity of 100% and specificity of 93% (Fig. 6; AUC = 0.98).

## Discussion

PS loosening is a main clinical concern after the spine fusion surgery. So, it has been reported that the various decision support systems or machine learning based models which are used to predict the pullout strength of pedicle screws. Tsai WC reported that the pullout strength of the pedicle screws was the functions of bone strength, screw design, and pilot hole using synthetic as specimens. And the PS loosening could be predicted using the various decision support systems [11].

However, the diagnosis of pedicle screw loosening after spine fusion surgery has been evaluated qualitatively by the presence of a radiolucent area and the halo sign surrounding the screw on plain X-rays and CT in general [8, 9]. In this study, we evaluated PS loosening quantitatively by measuring the displacement angle of the PS in the vertebrae while in a lying and a standing position using digital tomosynthesis. The results show that a displacement angle of at least 1.7° predicts screw loosening with a high sensitivity and specificity.

In a previous study, Emin et al. reported a quantitative method to diagnose PS loosening by measuring the change in angle between the PS axis and the end plate of the vertebra on plain radiographs at different postoperative timepoints. They suggested that a change of angle between the PS axis at the early postoperatively time and the next follow-up time, and the cranial endplate of at least 2° indicated PS loosening [3]. There are several limitations in the method of Emin et al. The influence of X-ray beam projection cannot be completely sorted out. They suggested that it is difficulty to acquire of the same slice images and to detect a unilaterally loosened screw [3].

Digital tomosynthesis can provide the same slice images while the patient is in lying and standing positions because of reconstruction within − 20° to 20° against the operating table. Because of its advantages, tomosynthesis can depict the unilateral PS on the same slice while the patient is in a lying and a standing position during the same period. Therefore, the new methods in this study are more accurate than those of previous reports. Previously we reported the usefulness of dynamic digital tomosynthesis radiculography to visualize the changes of nerve roots when the position of the patient is changed between prone and upright [12–14]. Furthermore, tomosynthesis can acquire images with a lower cost and lower radiation dose than CT. The radiation dose of tomosynthesis is twice that of radiographs and were reduced to approximately 1/10 of the CT dose [15].

The radiolucent zone and halo sign are thought to appear due to a decreased fit between the screw and bone. In a previous report, the presence of a radiolucent zone and halo sign surrounding the screw on plain X-ray and CT have been evaluated after at least 6 months postoperatively [9]. However, loosening of screws may occur during the early postoperative period (< 6 months) [16]. Mizuno et al. reported that rotational micromovement occurs between the PS and the vertebral body, and repeated rotational micromovements might cause loosening of the screw [17]. We hypothesized that the displacement of the screw in the vertebra might occur first through screw loosening. PS loosening might be detected during the early postoperative period by evaluating the displacement of the screw.

Movement between screw threads and bone inhibits bone formation, revascularization and remodeling of dead bone. Movement causes the screw to become enveloped by fibrous tissue in response to necrosis and leads to resorption of adjacent dead cortical bone. This results in a radiologically discernible radiolucent “halo” about the screw, a certain sign of screw loosening [18]. Early diagnosis of screw loosening can save the patient not only painful spasms but also possible serious neural damage or screw breakage [19].

In all imaging there is a phenomenon known as Mach effect or Mach bands [20]. So the presence or absence of the so called halo effect can be variable. The previous method of using the presence or absence of the “halo” was strongly influenced by the Mach effect. On the other hand, this new method of using the displacement angle can be unaffected by the Mach effect.

There are several limitations to our study. This method assumes that the shape of the vertebrae does not change between a lying and a standing position. However, the upper instrumented vertebra (UIV) fracture has often occurred after the spine fusion surgery. With the UIV fracture, the shape of vertebra changes in especially cranial and caudal endplate at the anterior part of the vertebra [21]. The posterior wall of the vertebral body is rarely damaged in UIV fractures. Therefore, the posterior wall is preserved. In this study, we evaluated all three angles at the cranial end of the fused segments. However, the posterior wall might be superior parameter in the identification of PS loosening.

The number of 10 pedicle screws (13%) in nine patients were excluded in the procedure due to issues with the plane of screw axis. The pedicle screw inserted inward by 20°or more was not evaluated because of reconstruction within − 20° to 20° against the operating table, in especially cases with lower lumbar spine and scoliosis.

The new method of evaluating PS loosening quantitatively demonstrated by this study might be helpful to detect PS loosening during the early postoperative period and decrease the risk of serious neural damage or revision surgery. This study might be useful in the evaluation of new spinal fusion instrumentation and the new technique of pedicle screw trajectory, such as penetrating endplate screw (PES). Furthermore, this method might be an alternative of CT and could evaluating the PS loosening in less radiation exposure than CT.

## Conclusion

The new quantitative method of diagnosing PS loosening using digital tomosynthesis in different postures is accurate. The optimal cut-off value of the PS displacement angle for identification of loosened screws is 1.7°.

## Data Availability

The datasets used and/or analysed during the current study are available from the corresponding author on reasonable request.

## Abbreviations

PS: Pedicle screw
CT: Computed tomography
ROC: Receiver Operating Characteristic
AUC: Area Under the Curve
PES: Penetrating endplate screw
UIV: Upper instrumented vertebra

## References

[1] Bokov A, Bulkin A, Aleynik A (2019). Peidicle screw loosening in patients with degenerative diseases of the lumbar spine: potential risk factors and relative contribution. Glob Spine J.

[2] Wu JC, Huang WC, Tsai HW, Ko CC, Wu CL, Tu TH, Cheng H (2011). Pedicle screw loosening in dynamic stabilization: incidence, risk, and outcome in 126 patients. Neurosurg Focus.

[3] Aghayev E, Zullig N, Diel P, Dietrich D, Lorin (2014). Benneker: development and validation of a quantitative method to assess pedicle screw loosening on posterior spine instrumentation on plain radiographs. Eur Spine J.

[4] Shea TM, Laun J, Gonzalez-Blohm SA, Doulgeris JJ, Lee WE 3rd, Aghayev K, et al. Designs and techniques that improve the pullout strength of pedicle screws in osteoporotic vertebrae: current status. Biomed Res Int. 2014:748393. 10.1155/2014/748393. 10.1155/2014/748393 PMC3958762 24724097

[5] Pearson HB, Dobbs CJ, Grantham E, Niebur GL, Chappuis JL, Boerckel JD (2017). Intraoperative biomechanics of lumbar pedicle screw loosening following successful arthrodesis. J Orthop Res.

[6] Galbusera F, Volkheimer D, Reitmaier S, Berger-Roscher N, Kienle A, Wilke H-J (2015). Pedicle screw loosening: a clinically relevant complication?. Eur Spine J.

[7] Bokov A, Bulkin A, Aleynik A, Kutlaeva M, Mlyavykh S (2019). Pedicle screws loosening in patients with degenerative diseases of the lumbar spine: potential risk factors and relative contribution. Glob Spine J.

[8] Sanden B, Olerud C, Petren-Mallmin M, Johansson C, Larsson S (2004). The significance of radiolucent zones surrounding pedicle screws. Definition of screw loosening in spinal instrumentation. J Bone Jt Surg Br.

[9] Tokuhashi, Matsuzaki, Oda, Uei (2008). Clinical Course and Significance of the Clear Zone Around the Pedicle Screws in the Lumbar Degenerative Disease. Spine (Phila Pa 1976).

[10] Okano E, Hara Y, Yamazaki M (2021). Novel method for selecting slices of the same cross-sectional view from digital tomosynthesis for monitoring for posterior spinal instrumentation. J Clin Neurosci.

[11] Tsai WC, Chen PQ, Lu TW, Wu SS, Shih KS, Lin SC (2009). Comparison and prediction of pullout strength of conical and cylindrical pedicle screws within synthetic bone. BMC Musculoskelet Disord.

[12] Mataki K, Koda M, Yamazaki M (2019). Successful visualization of dynamic change of lumbar nerve root compression with the patient in both upright and prone positions using dynamic digital tomosynthesis-radiculopathy in patients with lumbar foraminal stenosis: an initial report of three cases. J Clin Neurosci.

[13] Mataki K, Koda M, Yamazaki M (2020). New methods for diagnosing lumbar foraminal stenosis using dynamic digital tomosynthesis-radiculopathy. J Clin Neurosci.

[14] Mataki K, Koda M, Yamazaki M (2019). Dynamic digital tomosynthesis-radiculography is useful for diagnosis of lumbar foraminal stenosis at an adjacent level after lumbar fusion surgery: a case report. J Clin Neurosci.

[15] Noel A, Ottenin MA, Germain C (2011). Comparison of irradiation for tomosynthesis and CT of the wrist. J Radiol.

[16] Ninomiya I, Ohnishi O, Yoshimine. (2015). Clear zone formation around screws in the early postoperative stages after posterior lumbar fusion using the cortical bone trajectory technique. Asian spine J.

[17] Mizuno K, Sakakibara Y, Inaba. (2016). Biomechanical study of rotational micromovement of the pedicle screw. Springerplus.

[18] Schatzker J, Horne JG, Sumner-Smith G (1975). The effect of movement on the holding power of screws in bone. Clin Orthop Relat Res.

[19] Fras Dakhil-Jerew H, Jadeja AC, Shepperd JAN (2009). Inter-observer reliability of detecting Dynesys pedicle screw using plain X-rays: a study on 50 post-operative patients. Eur Spine J.

[20] Friedman AC, Lautin EN, Rothenberg L (1981). Mach bands and pneumomediastinum. J Can Assoc Radiol.

[21] Imai K (2015). Analysis of vertebral bone strength, fracture pattern, and fracture location: a validation study using a computed tomography-based nonlinear finite element analysis. Aging Dis.

